# Atypical emotional anticipation in high-functioning autism

**DOI:** 10.1186/s13229-015-0039-7

**Published:** 2015-08-15

**Authors:** Letizia Palumbo, Hollie G. Burnett, Tjeerd Jellema

**Affiliations:** Department of Psychological Sciences, University of Liverpool, Eleanor Rathbone Building, Bedford Street South, L69 7ZA Liverpool, UK; Department of Clinical Psychology, Medical School, University of Edinburgh, Teviot Place, EH8 9AG Edinburgh, UK; Department of Psychology, University of Hull, Cottingham Road, HU6 7RX Hull, UK

**Keywords:** High-functioning autism (HFA), Dynamic facial expressions, Perceptual distortions, Prediction, Anticipation, Embodied simulation, Theory of mind

## Abstract

**Background:**

Understanding and anticipating others’ mental or emotional states relies on the processing of social cues, such as dynamic facial expressions. Individuals with high-functioning autism (HFA) may process these cues differently from individuals with typical development (TD) and purportedly use a ‘mechanistic’ rather than a ‘mentalistic’ approach, involving rule- and contingency-based interpretations of the stimuli. The study primarily aimed at examining whether the judgments of facial expressions made by individuals with TD and HFA would be similarly affected by the immediately preceding dynamic perceptual history of that face. A second aim was to explore possible differences in the mechanisms underpinning the perceptual judgments in the two groups.

**Methods:**

Twenty-two adults with HFA and with TD, matched for age, gender and IQ, were tested in three experiments in which dynamic, ‘ecologically valid’ offsets of happy and angry facial expressions were presented. Participants evaluated the expression depicted in the last frame of the video clip by using a 5-point scale ranging from slightly angry via neutral to slightly happy. Specific experimental manipulations prior to the final facial expression of the video clip allowed examining contributions of bottom-up mechanisms (sequential contrast/context effects and representational momentum) and a top-down mechanism (emotional anticipation) to distortions in the perception of the final expression.

**Results:**

In experiment 1, the two groups showed a very similar perceptual bias for the final expression of joy-to-neutral and anger-to-neutral videos (overshoot bias). In experiment 2, a change in the actor’s identity during the clip removed the bias in the TD group, but not in the HFA group. In experiment 3, neutral-to-joy/anger-to-neutral sequences generated an undershoot bias (opposite to the overshoot) in the TD group, whereas no bias was observed in the HFA group.

**Conclusions:**

We argue that in TD individuals the perceptual judgments of other’s facial expressions were underpinned by an automatic emotional anticipation mechanism. In contrast, HFA individuals were primarily influenced by visual features, most notably the contrast between the start and end expressions, or pattern extrapolation. We critically discuss the proposition that automatic emotional anticipation may be induced by motor simulation of the perceived dynamic facial expressions and discuss its implications for autism.

## Background

The ability to interpret social cues conveyed by an agent as reflecting the agent’s mental or emotional state, allowing the observer to anticipate the agent’s behaviour, is essential for successful social interactions. In contrast to individuals with typical development (TD), individuals with autism spectrum disorders (ASD) are thought to have difficulties with this skill, or at least with its spontaneous employment [1, 2]. ASD is a pervasive developmental condition characterized by impaired social development and stereotypical, repetitive behaviours, which are often accompanied by obsessive interests and a lack of empathy [3–5]. High-functioning autism (HFA) is a mild form of autism with normal intelligence quotient (IQ) distribution, but with a delayed development of language skills and difficulties in social and emotional domains [6].

The extent to which atypical social and emotional behaviour in individuals with HFA reflects an impaired ability for implicit (automatic, non-volitional) rather than explicit (inferential, volitional, involving awareness) understanding is still unclear [7, 8]. Traditionally, the emphasis has been on explicit deficits in social understanding, such as the failure to attribute epistemic mental states (such as beliefs and desires) to others [9–11] or on failures in executive functioning [12]. However, more recently, impairments in implicit social processing increasingly gained attention, with suggestions that they may form a primary deficit [1, 2]. A failure in implicit or automatic understanding may be (partly) compensated by explicit reasoning on the basis of contextual information [2, 13].

A primary source of information on the basis of which implicit mental and emotional attributions can be formed is action sequences, either consisting of limb/body articulations or of changing facial expressions [14–16]. In humans, the perception of such simple actions, and indeed even single frames taken from the action sequence, has been shown to activate (pre)motor areas that are also involved in the execution of these actions, both with respect to limb actions [17] and facial expressions [18–21]. The matching of observation and execution of actions at the level of individual cells has been referred to as the mirror neuron mechanism (MNM) [22, 23]. The MNM may enable an automatic motor simulation of the observed action, which resembles the neuronal activity when the observer carries out the observed action him/herself. This may provide the observer with an appreciation of the other’s action ‘from within’, i.e. from a first person perspective rather than from a third person perspective [24]. Importantly, the motor simulation might additionally provide the observer with an automatic anticipation of what the other’s most likely next action or goal is. In other words, it may provide a view into the immediate future, on the basis of the immediate past. This idea is closely linked to the notion that action representations are neurologically arranged in action chains [15, 16, 25], where the chain specifies a goal. The automatic unfolding of the chain activity enables the automatic anticipation of the other’s next action or state of mind [26].

It is still much debated whether individuals with HFA have difficulties with action anticipation and whether this may be underpinned by impaired motor simulation. Difficulties in anticipating others’ actions in HFA have recently been suggested [27, 28]. Impaired motor simulation in ASD has been reported [29] but has also been contested [30].

### Using perceptual distortions to study emotional anticipation

The anticipation of actions consisting of dynamic facial expressions has recently been studied through a new task, which, in TD individuals, creates robust distortions in the perception of other’s facial expressions that are presumably due to anticipatory processes [31, 32]. This task involved the perception of short video clips of ‘ecologically valid’ dynamic facial expressions [33], in which intense happy or angry facial expressions morphed rapidly into neutral expressions (happy and angry offsets). The perceptual histories biased the evaluation of the neutral expressions, such that following a happy offset the neutral expression was evaluated as slightly angry, and following an angry offset as slightly happy. This was called an ‘overshoot’ response bias, involving a crossing of the category boundary. A series of manipulations were applied in order to clarify the nature of this bias [32]. For example, a change of facial identity just before the final neutral expression removed the overshoot bias [32]. This ruled out a major contribution of relatively low-level visual processes such as (sequential) contrast effects [34–36], adaptation [37, 38] and representational momentum (RM) [33, 39]. Sequential contrast/context effects were ruled out because the identity change did not alter the degree of contrast in emotion between the first and last expressions. Thus, if contrast was the driving force behind the overshoot bias, then the bias should not have been removed by the identity-change manipulation. Aftereffects induced by adaption to the initial facial expression (presented for 300 ms) were ruled out as they typically generalize across different identities. An explanation in terms of RM, which is the phenomenon that an observer’s memory for the final position of a moving target is displaced further along the observed trajectory, was also deemed unlikely. RM, when applied to dynamic facial expressions, could in principle operate on two distinct levels: (1) *The level of dynamic geometrical facial features* (*e.g. the U shape of the mouth in a smile turning flat and moving into a reversed U shape*). The changes in the facial expressions when going from one identity to the other are smooth enough not to disturb or reset RM. Indeed, when the identity transition was made really smooth by using morphing procedures [32], the TD group still did not report an overshoot bias in the identity-change condition; (2)* The level of an underlying valence dimension* (*a positive to negative dimension in the joy*-*to*-*neutral condition*, *and a negative to positive dimension in the anger*-*to*-*neutral condition*). However, the valence does not depend on the identity but on the expression, and therefore an identity change should not diminish RM.

Thus, extrapolations on both levels are unlikely explanations for the overshoot bias as neither should be affected by an identity change.

On the basis of these findings, the authors suggested that ‘emotional anticipation’, which is the involuntary and automatic anticipation of the other’s emotional state of mind on the basis of the immediate perceptual history, better explained the results. Emotional anticipation can be seen as a low-level mind-reading mechanism, enabling the tacit and intuitive understanding of another’s emotional state of mind [8]. Thus, in the joy-to-neutral videos, the observer implicitly anticipated the agent’s emotional state of mind to move into a ‘negative’ state, and in the anger-to-neutral videos to move into a ‘positive’ state. The idea is that these implicit ‘mentalizing’ activities in turn influenced perception [40].

### The current study

The current study explores the issue of whether emotion anticipation is impaired in HFA by utilizing the above paradigm [32]. The reasoning was that if individuals with HFA are impaired in the involuntary and automatic anticipation of the direction into which another’s emotional state develops, on the basis of the immediate dynamic perceptual history, then they would not report the visual distortion found in TD individuals. A number of experimental manipulations were introduced to examine whether the mechanisms underpinning perceptual report by the HFA group might be any different from those in the TD group. Individuals with HFA have been reported to use alternative or compensatory strategies, which may rely on physical or dynamical characteristics of the stimulus [27, 41]. In principle, any aberrant perceptual report in the HFA individuals might be due to either an over-reliance on contrast effects or RM, or to impaired emotional anticipation. The experimental manipulations were specifically designed to disentangle these mechanisms.

## Experiment 1

Short video clips of dynamic facial expressions, which morphed from 100 % joy or 100 % anger to either a neutral expression, a 10 % joy or a 10 % anger expression [32], were presented to the HFA and TD groups to assess any differences in their perceptual report of the facial expression displayed in the final frame. The primary hypothesis was that the individuals with HFA do not engage in ‘emotional anticipation’, which should result in the absence of an overshoot response bias. Alternatively, on the basis of findings suggesting that individuals with HFA can anticipate someone else’s actions by using alternative, compensatory strategies [27], it might be that they do show an overshoot bias but achieved it via a different route.

## Methods

### Participants

#### HFA group

Seventeen students with HFA participated in experiment 1. These formed a subset of the total of 22 individuals with HFA who took part in the three experiments comprising this study. The composition of the HFA groups differed slightly per experiment; group demographics are given for each experiment separately in Table 1.

**Table 1:** Demographic information for the HFA group and the TD group

	Group	*n*	Age	Sex	AQ	IQ-TOT	IQ-V	IQ-P	ADOS
Exp 1	HFA	16	20.3 (3.2)	5 F; 11 M	32.1 (7.4)	117.1 (10.9)	119.9 (14.7)	111.4 (14.7)	8.0 (0.9)
	TD	18	20.1 (4.0)	7 F; 11 M	15.3 (5.2)	113.4 (8.3)	114.1 (7.6)	111.8 (11.6)	
Exp 2	HFA	17	22.1 (7.2)	5 F; 12 M	29.0 (8.2)	115.9 (8.1)	117.2 (13.2)	112.1 (15.0)	8.1 (0.9)
	TD	17	22.1 (5.5)	6 F; 11 M	15.5 (6.6)	114.5 (7.7)	116.6 (6.4)	112.6 (12.6)	
Exp 3	HFA	15	22.3 (7.1)	5 F; 10 M	33.7 (5.7)	118.9 (8.9)	121.6 (13.6)	112.3 (11.9)	8.2 (1.6)
	TD	19	20.6 (2.5)	6 F; 13 M	14.7 (6.1)	114.7 (7.5)	115.5 (7.4)	112.5 (10.8)	

HFA and TD group characteristics in Experiments 1, 2 and 3. Age is in years. Standard deviations are shown between brackets

*F* female, *M* male, *AQ* Autism Spectrum Quotient, *IQ*-*T* total IQ score, *IQ*-*V* verbal IQ score, *IQ*-*P* performance IQ score, *ADOS* Autism Diagnostic Observation Schedule

The following description applies to all 22 HFA individuals. They all had previously received a diagnosis of HFA or Asperger syndrome from a clinical psychologist or psychiatrist based on DSM-IV-TR [42] or ICD-10 [4] criteria. All were recruited through disability services from universities in the north-east of England (UK). Evidence of diagnostic history for ASD was acquired, and in order to confirm ASD symptomology, the ADOS (Autism Diagnostic Observation Schedule, module 4) was completed with a trained examiner (HGB). The ADOS is a semi-structured, standardized assessment of communication, social interaction and imagination, designed for use with children and adults suspected of having ASD [43]. A score of 7–9 indicates autism spectrum, and a score of 10 or more indicates autism. The 22 individuals included in the study all met the ADOS criteria for HFA or Asperger syndrome (of the initial group, three individuals were excluded for having ADOS scores below 7). They also all completed the Autism Spectrum Quotient questionnaire (AQ) [44], which is a 50-statement, self-administered questionnaire, designed to measure the degree to which an adult with normal intelligence possesses autistic-like traits.

From the 17 students with HFA participating in experiment 1, one was removed following the application of exclusion criteria to the data (see ‘Data reduction’ below for details). The remaining 16 students (5 females, 11 males; mean age = 20.3 years, SD = 3.2) had a mean total ADOS score of 8.0 (SD = 0.9) and a mean AQ score of 32.1 (SD = 7.4). Their mean total IQ score was 117.1 (SD = 10.9; Table 1), assessed using the Wechsler Adult Intelligence Scale, WAIS-III [45].

#### TD group

All TD participants in the three experiments were undergraduate psychology students from Hull University. None of them took part in more than one experiment. All were asked if they had previously obtained a head injury or had received a diagnosis of ASD or of another mental health or developmental disorder. No participants disclosed this. Twenty TD individuals took part in experiment 1; applying the exclusion criteria to the data (see ‘Data reduction’ below) removed two individuals. The remaining 18 participants (7 females, 11 males; mean = 20.1 years, SD = 4.0) had a mean AQ score of 15.3 (SD = 5.2) and a mean total IQ score of 113.4 (SD = 8.3). The TD group did not differ from the HFA group in terms of age (*t*(32) = .554, *p* = .583), gender ratio (*X*^2^(1, 33) = .216, *p* = .642) or IQ (*t*(32) = −1.14, *p* = .264). As expected, AQ scores were significantly higher in the HFA group (*t*(32) = −7.74, *p* < .0001).

An important feature of the study was that the HFA group could be matched very closely with the control TD group, as both groups consisted of university students with fairly similar daily routines, resulting in a good approximation of the influence of the factor ‘HFA’. All HFA and TD participants had normal or corrected-to-normal vision and provided written consent prior to the experiment. Participants received course credits or a fee for taking part. The study was approved by the Ethics Committee of the Department of Psychology of Hull University.

#### Materials

The stimuli and experimental procedure were the same as in [32]. Pictures of eight actors displaying facial expressions of joy and anger were selected from the Pictures of Facial Affect (four males: EM, JJ, PE, WF; four females: C, MO, PF, SW) [46]. All faces were shown from frontal view with their eye gaze directed straight ahead. The photographs were in grayscale. The hair had been blackened so as to merge with the black background. The pictures were digitally adjusted to match in contrast and brightness. The eyes of all actors were positioned at approximately the same screen location. Faces measured about 13 × 20 cm when displayed on the screen, subtending approximately 8° vertically.

**Fig. 1:**
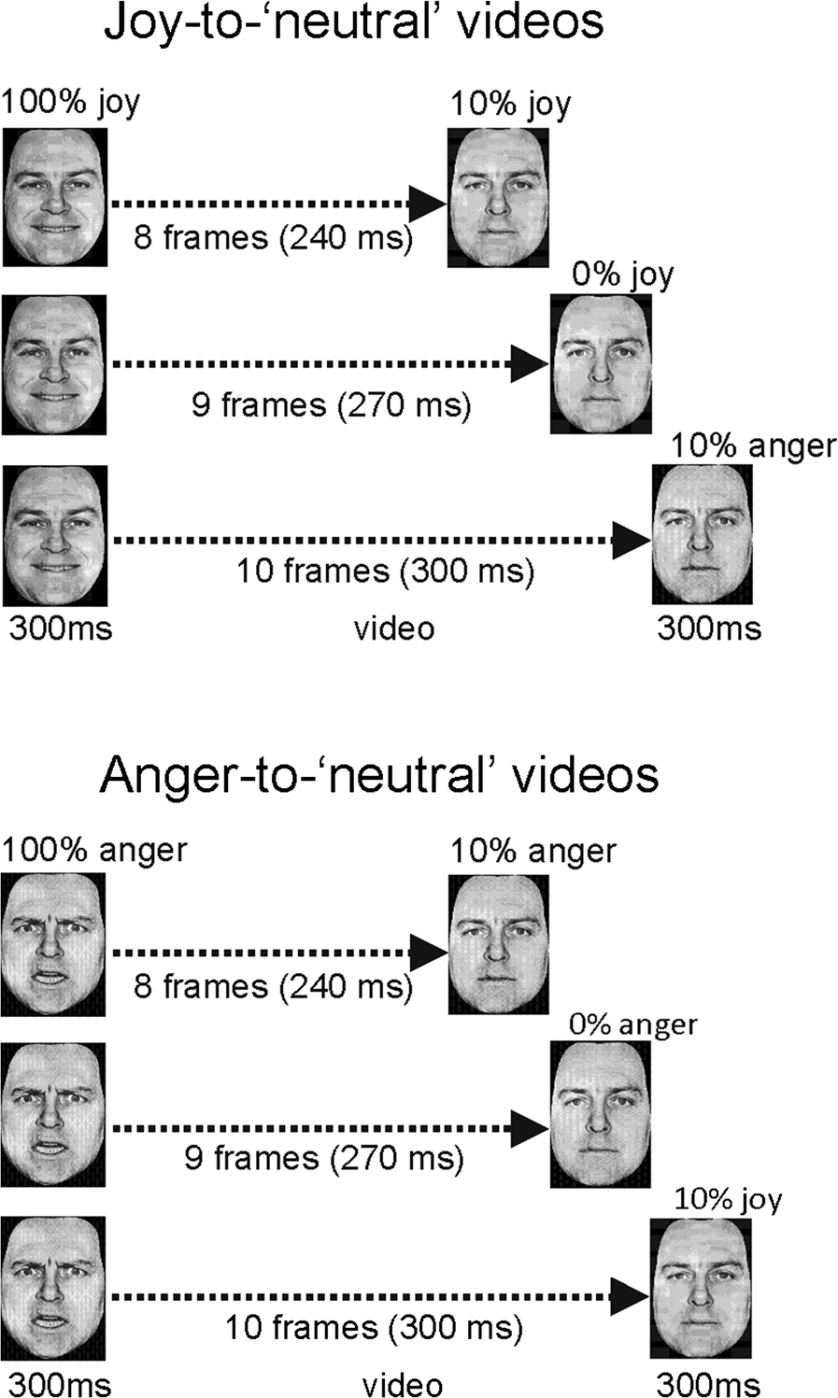
Illustration of the stimulus presentations in experiment 1. Joy-to-‘neutral’ videos started with a facial expression of intense joy, which gradually morphed into a 10 % joy, a neutral or a 10 % anger expression (*top panel*). Anger-to-‘neutral’ videos started with a facial expression of intense anger, which gradually morphed into a 10 % anger, a neutral or a 10 % joy expression (*bottom panel*)

Nine interpolated images, in between the full-blown expression of joy or anger (which is called 100 %) and the neutral expression (0 %), were created at equal steps of 10 % intensity change, using computer morphing procedures [47]. The resulting video clips depicted a maximally happy or angry expression of which the intensity gradually decreased until a neutral, a 10 % joy or a 10 % anger expression was reached. The first and the last frames each lasted 300 ms. The nine interpolated frames each lasted 30 ms (9 × 30 = 270 ms). The clips were 30 ms longer or shorter in case of 10 % intensity endpoints (Fig. 1).

#### Procedure

The experiment started with a calibration condition, in which the eight stimulus faces showed static neutral expressions (i.e. neutral expressions according to the ratings from [46]). Sixteen calibration trials were presented (eight actors, two repetitions each; random order) in which the neutral face was shown for 600 ms (preceded by a 500-ms fixation cross). Participants were prompted to rate the ‘neutral’ expressions using a 5-point scale, ranging from slightly angry (1) via neutral (3) to slightly happy (5), by pressing one of five labelled keys on a button box (SR-Box, Psychology Software Tools, Inc., USA).

Following the instructions displayed on the screen, eight practice trials were presented, in order to make sure that participants did understand the instructions and also to make them familiar with the task. The practice was followed by 64 experimental trials. Each trial started with a fixation cross (500 ms), directly followed by a video clip. As soon as the video ended, a blank screen appeared with a prompt to provide a response. The participant’s task was to evaluate the last expression using the same 5-point scale as used for the calibration trials. The 64 experimental trials were presented randomized, half of them starting with a happy expression (called joy-to-‘neutral’ videos) and half with an angry expression (called anger-to-‘neutral’ videos). Of both types of videos, 50 % of trials ended with the neutral expression, 25 % of trials ended with 10 % joy and 25 % of trials with 10 % anger expressions. The duration of the entire experimental procedure was 20 min.

#### Data reduction

Trials in which Reaction Times (RTs) were below 250 ms or above 3000 ms were considered outliers and were removed (HFA, 3.8 %; TD, 2.9 %). Participants were excluded if more than 25 % of their RT values fell outside the above range (HFA, *n* = 0; TD, *n* = 1) and when they pressed the same key for more than 90 % of trials (HFA, *n* = 0; TD, *n* = 0). A ±2.5 SD rule was applied to the mean difference of the ratings per participant, i.e. rating in the anger-to-neutral condition minus rating in the joy-to-neutral condition (HFA, *n* = 1; TD, *n* = 1).

For both groups, the mean calibration scores obtained for each of the eight stimulus faces were used to adjust the scores in the experimental trials; a calibration factor [equal to 3.00 minus the calibration score] was added to the experimental scores. The calibration procedure allowed performing one-sample *t* tests with test value 3.00 on the scores in the two perceptual histories. All statistical analyses were performed on the calibrated scores.

## Results

**Fig. 2:**
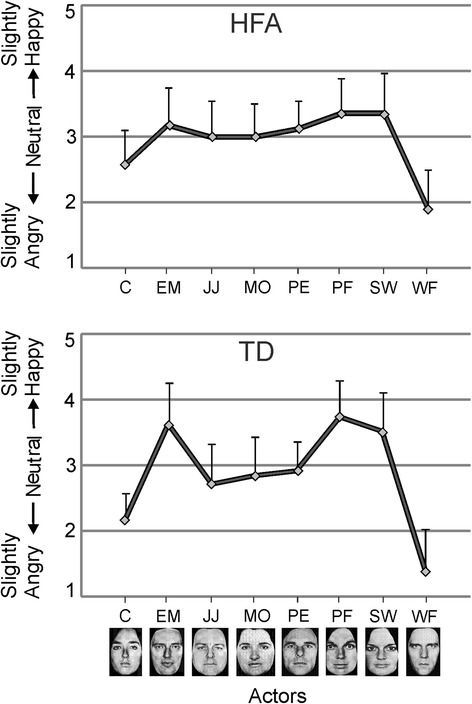
Calibration scores in experiment 1. Ratings on the 5-point scale (*vertical axis*) for the neutral expression. Scores are shown for each of the eight actors for HFA (*top panel*) and TD individuals (*bottom panel*). *Error bars* indicate +1SD

An illustration of the scores in the calibration phase for the neutral expressions of each actor in the TD and HFA groups is shown in Fig. 2. The calibration scores were similar between the two groups, with the neutral expression of actors C and WF consistently rated as slightly angry.

**Fig. 3:**
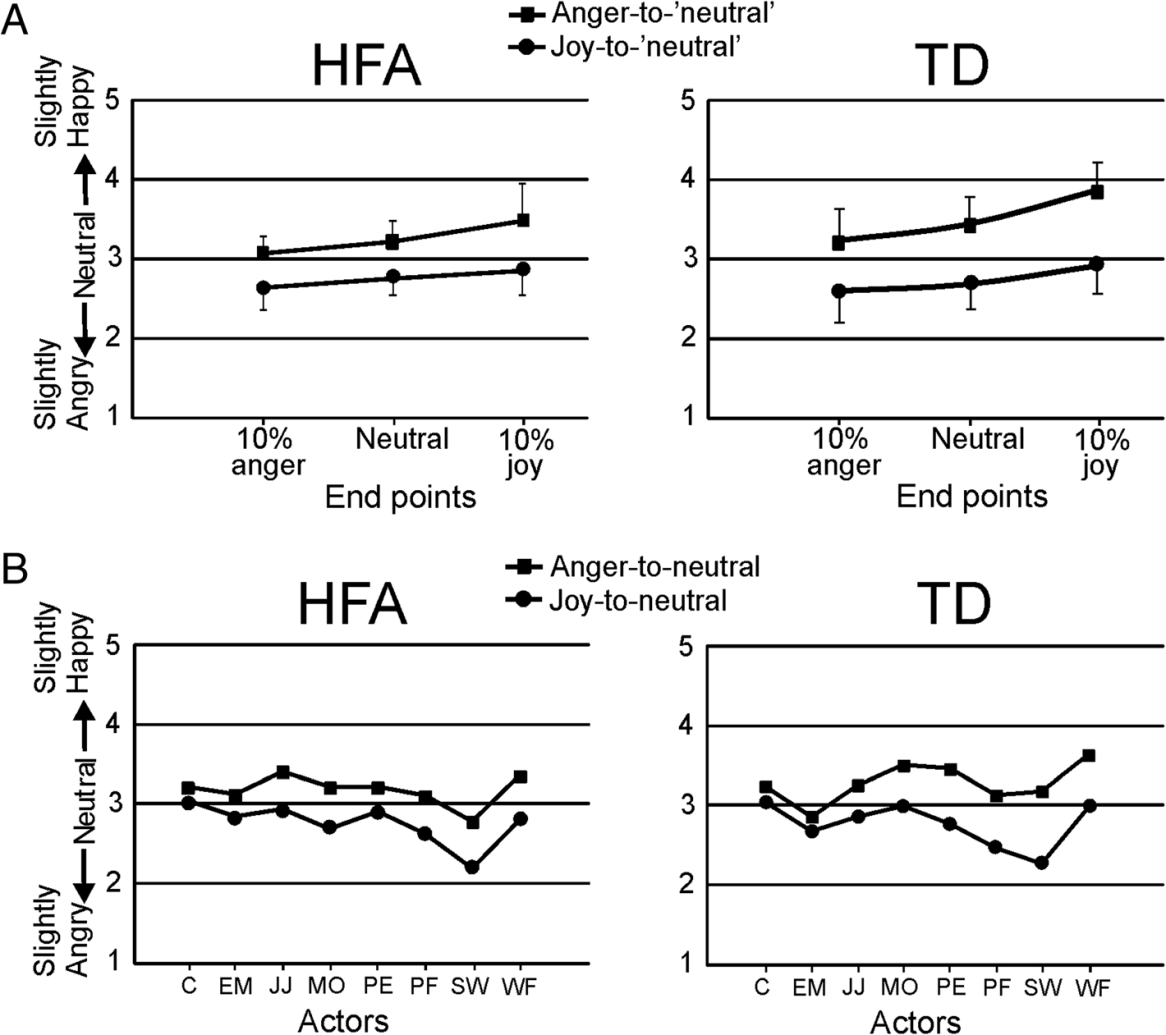
Results of experiment 1. **a** Ratings on the 5-point scale for the expressions depicted in the last frame of the joy-to-‘neutral’ and anger-to-‘neutral’ videos for the HFA group (*left side*) and the TD group (*right side*). The sequences ended at 10 % anger, neutral or 10 % joy. *Error bars* indicate 1SD. **b** Ratings for exclusively the neutral expressions at the end of the happy and angry videos for the HFA and TD groups. Scores are shown for each of the eight actors to illustrate consistency

The results of the experimental trials are illustrated in Fig. 3. A 3 x 2 x 2 repeated measures ANOVA was performed with the endpoint of the video (10 % anger vs. neutral vs 10 % joy) and perceptual history (joy-to-'neutral' videos vs. anger-to-'neutral' videos), and group as between-subjects factor (HFA vs. TD). The main effect of endpoint was significant (*F*(2, 64) = 43.9, *p* < .0001, *η*_*p*_^2^ = .58), reflecting that when the videos ended at 10 % joy, the expressions in the last frame were judged as more happy than when they ended at neutral (*t*(15) = −4.14, *p* = .001) or at 10 % anger (*t*(15) = 4.54, *p* < .001), while the 10 % anger and neutral endpoints did not differ from each other (*t*(15) = 2.50, *p* = .024; *α* = .017 after correction for multiple comparisons). The main effect of perceptual history was significant (*F*(1, 32) = 223.8, *p* < .0001, *η*_*p*_^2^ = .88), reflecting that the final expressions were judged as more angry in the joy-to-neutral condition than in the anger-to-neutral condition. There was no significant main effect of group (*F*(1, 32) = 2.1, *p* = .15, *η*_*p*_^2^ = .063).

The endpoint by group interaction was non-significant (*F*(2, 64) = .94, *p* = .40, *η*_*p*_^2^ = .029), nor was the perceptual history by group interaction (*F*(1, 32) = 3.3, *p* = .078, *η*_*p*_^2^ = .095). The endpoint by perceptual history interaction was significant (*F*(2, 64) = 4.1, *p* = .021, *η*_*p*_^2^ = .11), reflecting that across both groups the difference between the judgments in the two perceptual histories at the 10 % joy endpoint was larger than at the neutral (*t*(33) = 2.7, *p* = .030) and 10 % anger (*t*(33) = 2.6, *p* = .013) endpoints. The three-way interaction was non-significant (*F*(2, 64) = .063, *p* = .94, *η*_*p*_^2^ = .002).

The judgments made at the neutral video endpoint were examined in more detail, as this endpoint, due to being neither positive nor negative, is most sensitive to a misattribution of emotional content involving a crossing of the category boundary. In the HFA group, the mean ratings in the joy-to-neutral videos (*M* = 2.73, SD = 0.22) and anger-to-neutral videos (*M* = 3.21, SD = 0.28) differed significantly from each other (*t*(15) = −6.94, *p* < .0001), and each of them differed significantly from 3.00 (joy video, *p* < .0001; anger video, *p* = .010). The TD group showed a very similar pattern; the mean ratings in the joy-to-neutral videos (*M* = 2.71, SD = 0.27) and anger-to-neutral videos (*M* = 3.36, SD = 0.31) differed significantly from each other (*t*(17) = −7.04, *p* < .0001), and each of them differed significantly from 3.00 (joy video, *p* < .001; anger video, *p* < .0001).

## Discussion

Both HFA and TD participants showed a robust overshoot response bias, with the last expression of the joy-to-neutral videos evaluated as slightly angry and the identical last expression of the anger-to-neutral videos evaluated as slightly happy. The main conclusion is that the evaluations of both groups were influenced by the perceptual histories, and to roughly the same extent (with a tendency for the TD group to be influenced more than the HFA group). The results seem to be in line with a study by [48] who reported that a group of individuals with developmental pervasive disorders overestimated the intensity of facial expressions in neutral-to-emotional expression video clips similar to a TD group, although to a lesser extent.

If the HFA group had shown no overshoot bias, then that would have suggested that they do not engage in an ‘emotional anticipation’ process. The presence of the overshoot bias leaves open two possibilities: (1) the HFA group engages in involuntary emotional anticipation similar to the TD group, and (2) they rely on another mechanism, which compensates for the absence of automatic emotional anticipation. Experiments 2 and 3 were conducted to clarify this issue. Specifically, they examined whether individuals with HFA may have processed the dynamic facial expressions predominantly in terms of physical characteristics, without keeping track of the changes in the agent’s emotional state of mind that cause the changes in facial expression.

## Experiment 2

Experiment 2 was conducted to test whether low-level visual mechanisms, underpinning contrast and RM effects, may explain the overshoot effect found in experiment 1 in the HFA group. Hereto, the identity of the actor was changed in the last frame of the clips, similar to the manipulation performed in [32]. Thus, participants needed to judge the last expression of the videos depicting a new identity for which no immediate perceptual history was available. The rational was that if the overshoot bias in HFA individuals resulted from sequential contrast effects or from RM operating on an underlying positive–negative valence dimension, rather than from emotional anticipation, then a change in identity should have no effect. A change in the agent’s identity does not affect the contrast between the first and last frames nor does it interfere with the underlying positive–negative valence dimension. On the other hand, if the bias in the HFA group did result from emotional anticipation, then they should, similar to the TD group in [32], not show an overshoot response bias. After all, one cannot anticipate the emotional state of someone on the basis of a perceptual history pertaining to a different identity.

### Methods

#### Participants

##### HFA group

Twenty individuals with HFA took part in experiment 2, of which three were removed following the application of data exclusion criteria (see ‘Data reduction’ below). The remaining 17 individuals (5 females, 12 males; mean age = 22.1 years, SD = 7.2) had a mean total ADOS score of 8.1 (SD = 0.9) and a mean AQ score of 29.0 (SD = 8.2). Their mean total IQ score was 115.9 (SD = 8.1; WAIS-III; Table 1).

##### TD group

Twenty-one TD individuals took part. Applying the data exclusion criteria removed four participants. The remaining 17 participants (6 females, 11 males; mean age = 22.1 years, SD = 5.5) had a mean AQ score of 15.5 (SD = 6.6) and a mean total IQ score of 114.5 (SD = 7.7). The TD group did not differ significantly from the HFA group in terms of age (*t*(32) = .675, *p* = .10), gender ratio (*X*^2^(1, 34) = .134, *p* = .714) or IQ (*t*(32) = −.54, *p* = .591). AQ scores were significantly higher in the HFA group (*t*(32) = 5.28, *p* < .0001).

All HFA and TD participants had normal or corrected-to-normal vision and provided written consent prior to the experiment. Participants received course credits or a fee for taking part. The study was approved by the Ethics Committee of the Department of Psychology of Hull University.

#### Materials

**Fig. 4:**
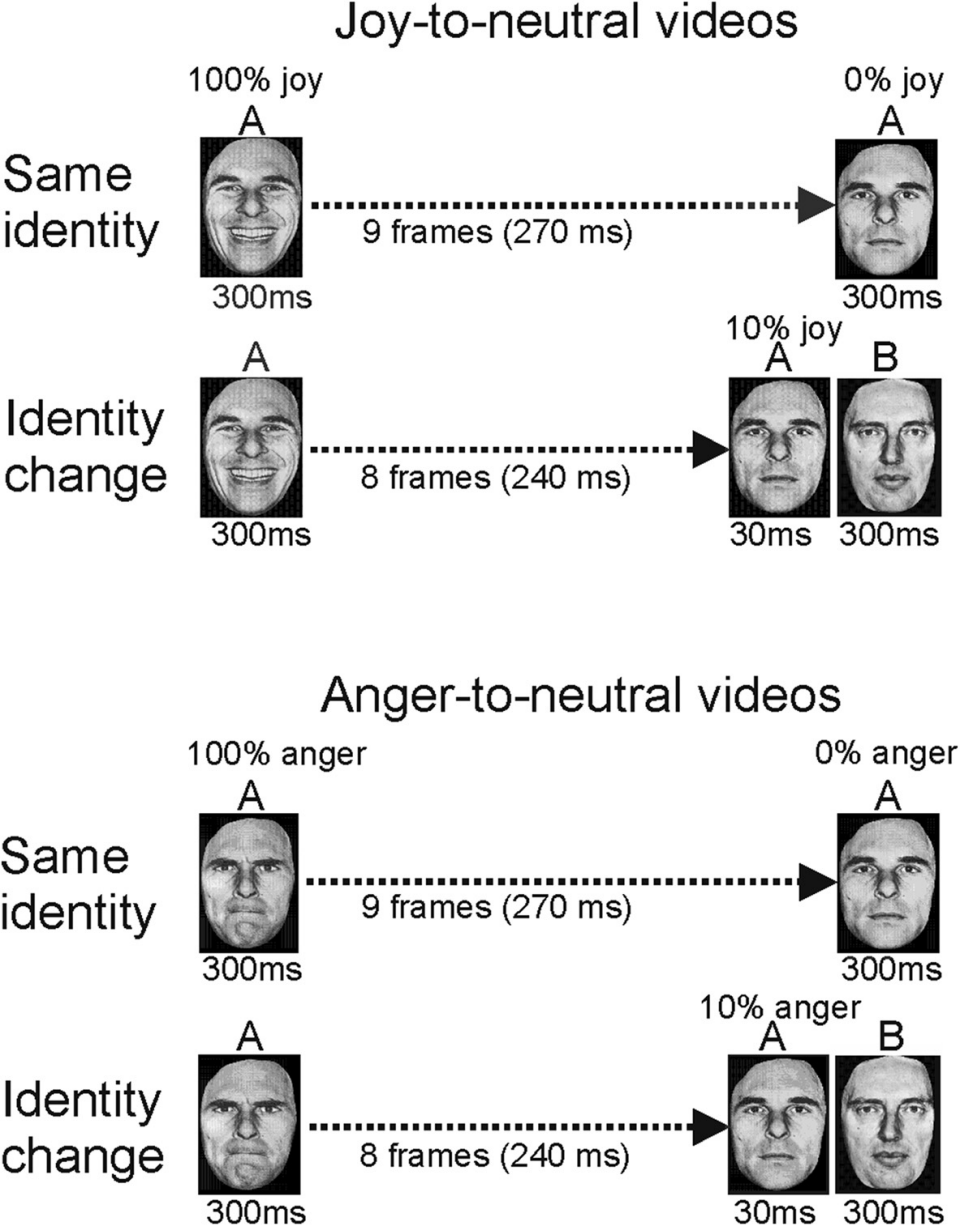
Illustration of the stimulus presentations in experiment 2. Shown are the same-identity condition and the identity-change condition, for joy-to-neutral and anger-to-neutral perceptual histories

The control condition (same identity) involved the same videos as used in experiment 1, be it that exclusively videos ending at neutral expressions were used. In the identity-change condition, the identity of the actor changed instantly in the last frame of the sequence (Fig. 4). Both same gender and different gender changes were included.

#### Procedure

The procedure was the same as for experiment 1. Following the calibration phase and the practice trials, 128 experimental trials (8 actors × 2 perceptual histories × 2 conditions × 4 repetitions) were presented. The same-identity and identity-change conditions were presented in separate blocks (each 64 trials).

#### Data reduction

Exclusion criteria were the same as for experiment 1. Trials in which RTs were below 250 ms or above 3000 ms were removed (HFA, 2.3 %; TD, 5.7 %). Participants were excluded if more than 25 % of their RT values fell outside the above range (HFA, *n* = 0; TD, *n* = 2) and when they pressed the same key on more than 90 % of trials (HFA, *n* = 1; TD, *n* = 0). The ±2.5 SD rule was applied to the mean difference of the ratings in the ‘joy’ and ‘anger’ conditions per participant (HFA, *n* = 2; TD, *n* = 2).

### Results

**Fig. 5:**
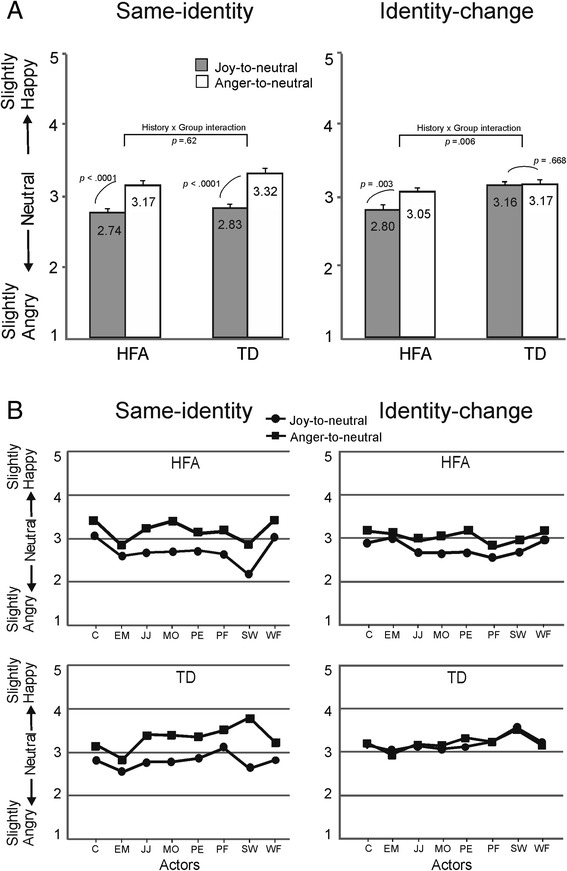
Results of experiment 2. **a** Scores for the neutral expressions at the end of the joy-to-neutral and anger-to-neutral sequences in the same-identity (*left side*) and identity-change (*right side*) conditions in HFA and TD individuals. *Error bars* indicate SEM. **b** Ratings for each of the eight actors in the same-identity (*left side*) and identity-change (*right side*) conditions are shown to illustrate the consistency across actors in HFA and TD individuals

The scores in the calibration phase were very similar to those of experiment 1. Initially, an overall 2 × 2 × 2 ANOVA was performed with task (same identity vs. identity change) and perceptual history (joy-to-neutral vs. anger-to-neutral) as within-subject factors, and group (HFA vs. TD) as between-subjects factor. The main effect of task was non-significant (*F*(1, 32) = 1.02, *p* = .32, *η*_*p*_^2^ = .031). The main effect of perceptual history was highly significant (*F*(1, 32) = 65.9, *p* < .0001, *η*_*p*_^2^ = .67), reflecting the overshoot response bias. The main effect of group was also significant (*F*(1, 32) = 1.05, *p* = .010, *η*_*p*_^2^ = .19), with the HFA group scoring lower (i.e. more ‘anger’ judgments) than the TD group. The task by perceptual history interaction factor was significant (*F*(1, 32) = 28.9, *p* < .0001, *η*_*p*_^2^ = .48), while the other two-way interaction factors were not significant (task by group: *F*(1, 32) = 3.8, *p* = .060, *η*_*p*_^2^ = .106; perceptual history by group: *F*(1, 32) = 1.44, *p* = .24, *η*_*p*_^2^ = .04). The three-way interaction factor was significant (*F*(1, 32) = 5.4, *p* = .027, *η*_*p*_^2^ = .14). Next, the two tasks were analysed separately (Fig. 5).

#### Same-identity task

A 2 × 2 ANOVA showed a significant main effect of perceptual history (*F*(1, 32) = 70.4, *p* < .0001, *η*_*p*_^2^ = .68). The main effect of group was non-significant (*F*(1, 32) = 3.23, *p* = .082, *η*_*p*_^2^ = .092), and the perceptual history by group interaction was non-significant (*F*(1, 32) = .25, *p* = .62, *η*_*p*_^2^ = .008). Thus, the results of the TD and HFA groups on the same-identity task replicated those of experiment 1, both showing a robust overshoot response bias.

#### Identity-change task

The 2 × 2 ANOVA showed significant main effects of both perceptual history (*F*(1, 32) = 11.17, *p* = .002, *η*_*p*_^2^ = .259) and group (*F*(1, 32) = 10.02, *p* = .003, *η*_*p*_^2^ = .239). Crucially, the perceptual history by group interaction was significant (*F*(1, 32) = 8.70, *p* = .006, *η*_*p*_^2^ = .214). In the TD group, the ratings for the neutral expressions did not differ between the two perceptual histories (*t*(16) = .437, *p* = .67). Thus, in TD participants, a change of identity of the actor effectively removed the overshoot bias. In the AS group, however, the overshoot bias persisted (*t*(16) = 3.52, *p* = .003). While in the same-identity condition the two groups performed almost identically, in the identity-change condition the groups differed significantly, with the HFA group, but not the TD group, showing an overshoot response bias. This was also clearly apparent when the results are shown for each actor separately (Fig. 5b).

#### Could impaired identity recognition skills in the HFA group explain their overshoot bias in the identity-change condition?

**Fig. 6:**
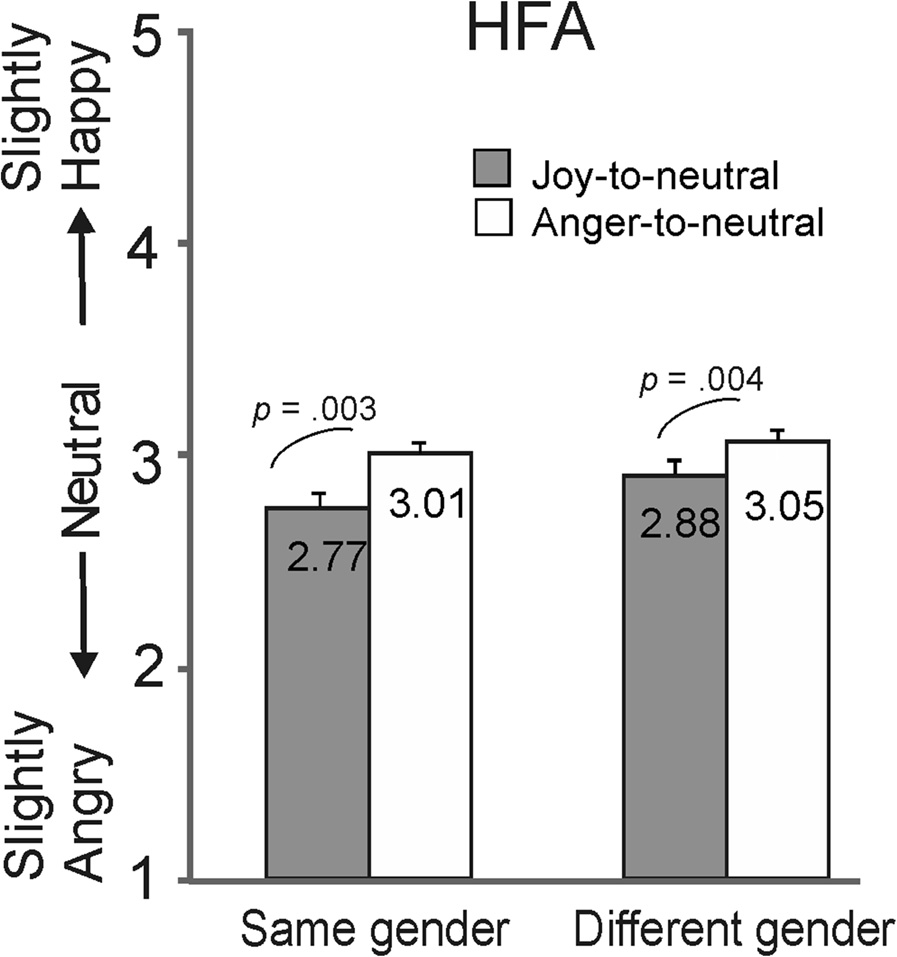
The effect of gender switch in experiment 2. Scores for the neutral expressions at the end of the joy-to-neutral and anger-to-neutral sequences are shown in the identity-change condition with the same gender change and different gender change in HFA individuals

In principle, the overshoot bias found in the identity-change condition in HFA individuals might have resulted from impaired identity recognition skills [49]. Even though from debriefing HFA participants it emerged that they all had spotted the identity change, we could not be sure that this was the case in each and every trial. If the HFA participants had not detected the identity change in some of the trials, then they would have treated these trials as if they were same-identity trials. We reasoned that if this was the case, then more overshoot was expected in trials where the two identities had the same gender, compared to trials with a gender shift. In terms of physical features, a male and a female face would look more dissimilar than two male or two female faces. Therefore, in a subsequent analysis of the HFA data, the same gender and different gender conditions were separated. In both the same gender and different gender conditions, the mean scores in the joy-to-neutral and anger-to-neutral videos differed significantly from each other (both *p*s < .005; paired samples *t* tests, see Fig. 6). The mean difference scores [i.e. the mean score in anger-to-neutral videos minus the mean score in joy-to-neutral videos] in the trials with and without a gender switch did not differ (*t*(16) = 1.23, *p* = .237). This suggests that poorer identity recognition skills did not play a role in bringing about the overshoot bias in the identity-change condition in the HFA group.

### Discussion

When the video clips contained no change in identity, both HFA and TD individuals produced a robust overshoot response bias, confirming the results of experiment 1. However, following the identity-change manipulation, the overshoot bias persisted in the HFA group but was absent in the TD group. Separate analysis of same gender and different gender trials in the identity-change condition suggested that this most probably could not be explained by impaired identity recognition in the HFA group.

Whereas the results of the same-identity condition might suggest that HFA individuals show emotional anticipation, similar to TD individuals, the results of the identity-change condition show that this is probably not the case. Emotional anticipation would not produce an overshoot bias in response to a new agent B the observer knows nothing about (i.e. no perceptual history), other than that agent B depicts a neutral expression. These findings open up the possibility that the HFA group relied on an alternative mechanism or strategy to produce the bias in the same-identity condition and that the same mechanism/strategy was applied in the identity-change condition, hence their bias in the latter condition.

One possibility is that the HFA group might have been influenced by the contrast in facial expressions between the 100 % emotional expression shown at the start of the clip and the neutral expression shown at the end of the clip (both presented for 300 ms). RM [39, 50], involving an extrapolation of the dynamic geometries in the face or an extrapolation on an underlying positive–negative valence dimension, forms in principle another possible explanation. The underlying positive–negative valence dimension and to a large extent also the extrapolation of the dynamic geometries of the face remain unaffected by an identity change.

The absence of an overshoot bias in the TD group in the identity-change condition confirmed the findings reported in [32]. It suggests that the robust overshoot in the same-identity condition was not produced by sequential contrast effects or RM, because if TD participants relied on these low-level, bottom-up processes, then they should also have been affected by them in the identity-change condition. This finding favours a top-down emotional anticipation explanation for the TD group.

## Experiment 3

In experiment 3, the video sequence was modified such that it started from a neutral expression and morphed via a maximally happy or angry expression back to the same neutral expression (loop condition). The loop task was designed to shed light on the merits of RM [33, 39] and contrast effects [34–36] as an explanation for the overshoot response bias in the HFA group. The rational was that if the response bias found in the previous experiments in HFA individuals was induced by the contrast between the to-be-evaluated last neutral expression (presented for 300 ms) and the ‘anchor’ stimulus (the first happy or angry expression, also presented for 300 ms), then in the loop condition, one would expect this last neutral expression to be evaluated as neutral and not to obtain an overshoot bias. Alternatively, RM would predict to find an overshoot response bias in the loop condition, i.e. an extrapolation of the second half of the sequence that went from maximal joy/anger to neutral. In the study by Palumbo and Jellema [32], the TD group produced an ‘undershoot’ effect in the loop condition, consisting of a bias in the direction of the immediately preceding emotion (i.e. a bias towards happy in the neutral-to-happy-to neutral condition and a bias towards angry in the neutral-to-angry-to-neutral condition).

### Methods

#### Participants

##### HFA group

Eighteen individuals with HFA took part, of which three were removed following the application of data exclusion criteria (see ‘Data reduction’ below). The remaining 15 individuals (5 females, 10 males; mean age = 22.3 years, SD = 7.1) had a mean total ADOS score of 8.2 (SD = 1.6), a mean AQ score of 33.7 (SD = 5.7) and a mean total IQ score of 118.9 (SD = 8.9; WAIS-III; Table 1).

##### TD group

Twenty-two TD individuals took part. Applying the data exclusion criteria removed three participants. The remaining 19 individuals (6 females, 13 males; mean age = 20.6 years, SD = 2.52) had a mean AQ score of 14.7 (SD = 6.1) and a mean total IQ score of 115.1 (SD = 8.1).

The TD group did not differ from the HFA group in terms of age (*t*(32) = 1.00, *p* = .323), gender ratio (*X*^2^(1, 34) = .012, *p* = .914) or IQ (*t*(32) = 1.49, *p* = .147). AQ scores were significantly higher in the HFA group (*t*(32) = 9.29, *p* < .0001).

All HFA and TD participants had normal or corrected-to-normal vision and provided written consent prior to the experiment. Participants received course credits or a fee for taking part. The study was approved by the Ethics Committee of the Department of Psychology of Hull University.

#### Materials

**Fig. 7:**
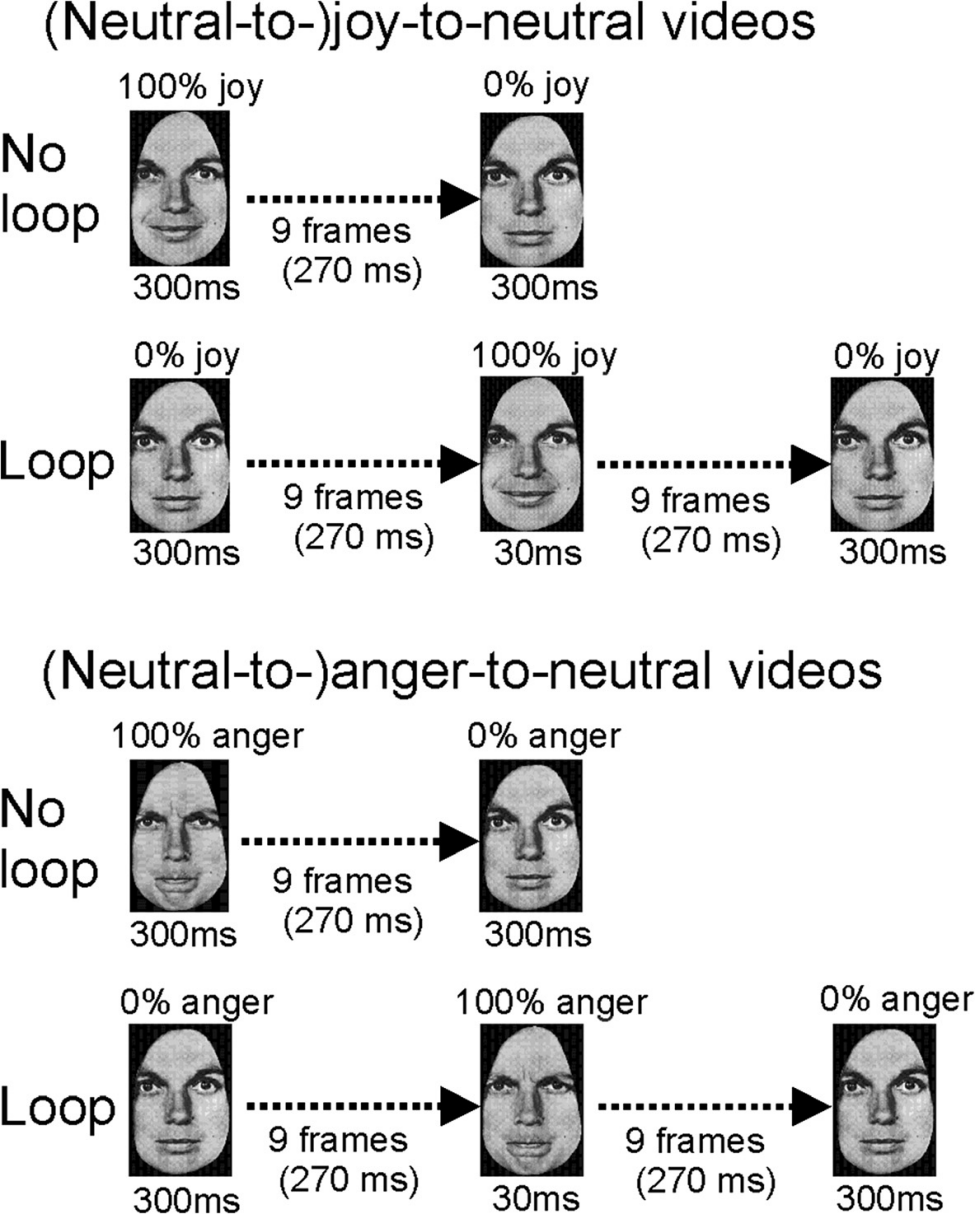
Illustration of the stimulus presentations in experiment 3. *Top panel*: Joy-to-neutral sequence in the control condition (*top row*) and neutral-to-joy-to-neutral sequence in the loop condition (*bottom row*). *Bottom panel*: Similar sequences but for anger-to-neutral (*top row*) and neutral-to-anger-to-neutral (*bottom row*)

Video clips displayed a neutral expression, which morphed via a maximally happy or angry expression back to the same neutral expression. The morphing sequence consisted of 19 interpolated frames, each 30 ms long. The first and the last frames both lasted 300 ms, making the entire sequence last for 1170 ms. The control condition (no loop) consisted of joy-to-neutral and anger-to-neutral video sequences (Fig. 7).

#### Procedure

Following the calibration and the practice trials as in the previous experiments, participants were presented with 96 randomized trials: 8 actors × 2 perceptual histories × 2 conditions × 3 repetitions.

#### Data reduction

Trials in which RTs were below 250 ms or above 3000 ms were removed (HFA, 9.2 %; TD, 7.6 %). Participants were excluded if more than 25 % of their RT values fell outside this range (HFA, *n* = 0; TD, *n* = 2) and for pressing the same key more than 90 % of trials (HFA, *n* = 1; TD, *n* = 0). A ±2.5 SD rule was applied to the mean difference of the ratings in the ‘joy’ and ‘anger’ conditions per participant (HFA, *n* = 2; TD, *n* = 1).

### Results

**Fig. 8:**
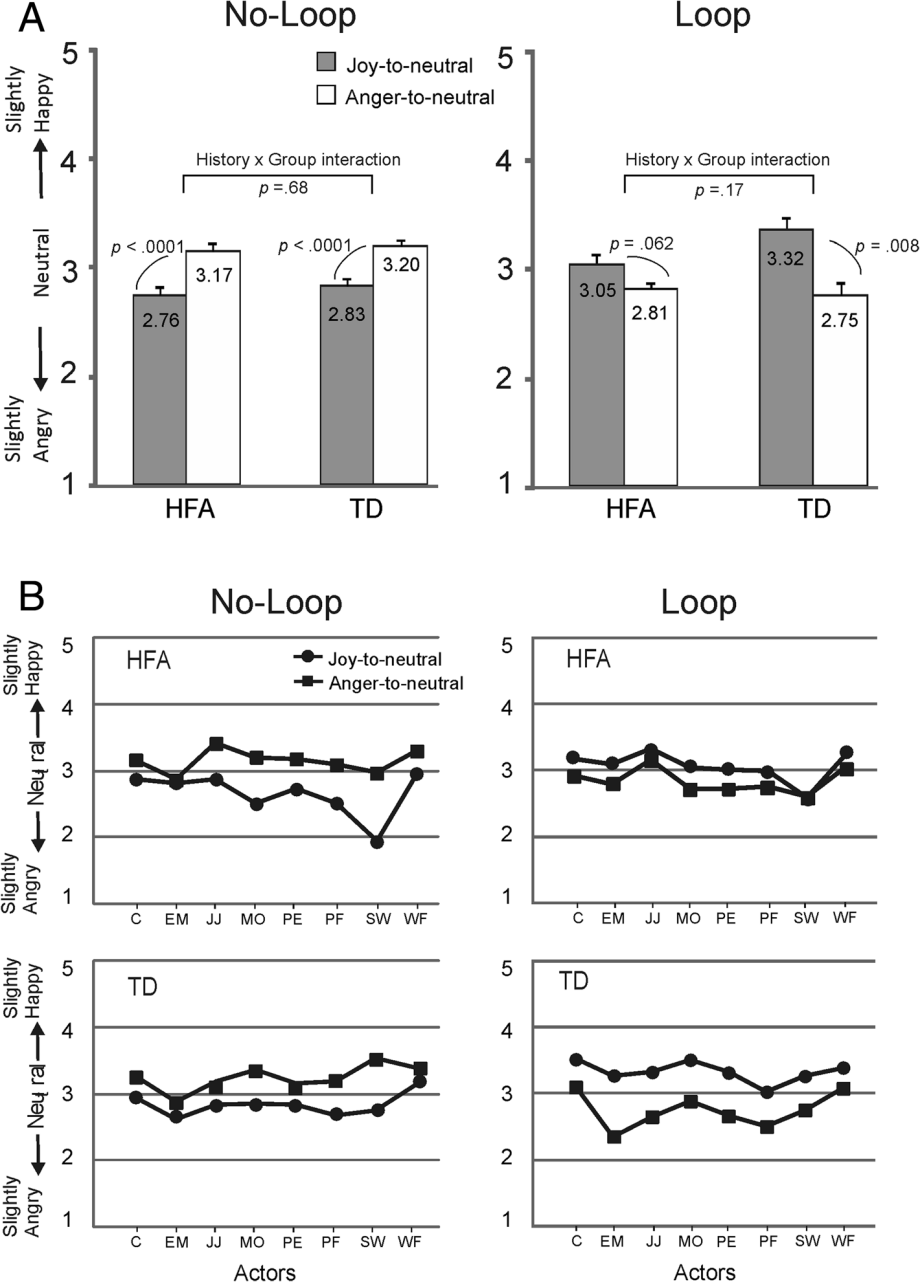
Results of experiment 3. **a** Scores for the neutral expressions at the end of the joy-to-neutral and anger-to-neutral sequences in the no-loop (*left side*) and loop (*right side*) conditions for the HFA and TD groups. *Error bars* indicate SEM. **b** Ratings for each of the eight actors in the no-loop and loop conditions are shown to illustrate the consistency across actors for the HFA and TD groups

A 2 × 2 × 2 ANOVA was performed with task (no loop vs. loop) and perceptual history (joy-to-neutral vs. anger-to-neutral) as within-subject factors, and group (TD vs. HFA) as between-subjects factor. The main effect of task was non-significant (*F*(1, 32) = .038, *p* = .85, *η*_*p*_^2^ = .001). The main effect of perceptual history was also non-significant (*F*(1, 32) = .042, *p* = .84, *η*_*p*_^2^ = .001). The latter was surprising as so far the main effect of perceptual history had always been highly significant. It reflected that in the loop task, the pattern of scores was effectively the opposite of that in the no-loop task (Fig. 8). The main effect of group was non-significant (*F*(1, 32) = 1.4, *p* = .24, *η*_*p*_^2^ = .042). Of the two-way interaction factors, the task by perceptual history interaction was significant (*F*(1, 32) = 35.1, *p* < .0001, *η*_*p*_^2^ = .52), reflecting the pattern reversal between the two tasks. The other two-way interaction factors were non-significant (task by group: *F*(1, 32) = .932, *p* = .34, *η*_*p*_^2^ = .028; perceptual history by group: *F*(1, 32) = 2.08, *p* = .16, *η*_*p*_^2^ = .061). The three-way interaction was non-significant (*F*(1, 32) = 1.23, *p* = .28, *η*_*p*_^2^ = .037). Even though there was no significant three-way interaction, we analysed the group differences further in the loop and no-loop conditions for purely explorative purposes using *t* tests (with *α* set at .0125 to correct for multiple comparisons). This explorative analysis suggested that in the loop task, the ‘undershoot’ bias in the HFA group did not reach significance (*t*(14) = 2.03, *p* = .062), while in the TD group, it did (*t*(18) = 2.97, *p* = .008).

### Discussion

In the control condition (no loop), the HFA and TD groups showed a very similar overshoot response bias, confirming the results of experiments 1 and 2. In the loop task, both the TD and HFA groups showed the reversed pattern, i.e. a tendency towards an undershoot response bias: the last frame of the neutral-to-joy-to-neutral sequence was evaluated as ‘slightly happy’ and the last frame of the neutral-to-anger-to-neutral sequence as slightly angry. This confirmed previous findings in [32].

In neither group, an overshoot bias was found in the loop task, suggesting that RM was not the underpinning mechanism for either group. The sequential contrast hypothesis predicted that the last frame would be evaluated as neutral, as the first and last frames were identical (both neutral). The tendency towards an undershoot response in both groups renders a major contribution of sequential contrast unlikely. However, the explorative analysis suggested that the undershoot bias in the HFA group was rather weak; its reliability will have to be determined by further testing. An absence of a bias in the loop condition in the HFA group might suggest that their responses were determined by sequential contrast effects, which would open up the possibility that their overshoot biases in the control condition were also induced by sequential contrast effects, rather than by emotional anticipation.

A possible explanation for the undershoot response in the TD group is that the neutral-joy-neutral sequence conveyed a positive social signal directed towards the observer (a brief smile, as used e.g. when colleagues acknowledge each other while passing in the corridor). Similarly, the neutral-anger-neutral sequence could convey a negative social signal. This induced in the observer the impression that the agent either liked or disliked them, and, as we tend to like those who like us and dislike those who dislike us [51], the agent was evaluated accordingly. It is important to note that susceptibility to the ‘social signal’ does not necessarily imply that the observer is capable of ‘emotional anticipation’.

## General discussion

The current study examined whether individuals with HFA show similar or dissimilar biases to TD individuals in their evaluations of dynamic facial expressions, and whether the mechanisms they rely on in making these evaluations might be any different from those of TD individuals. Palumbo and Jellema [32] suggested that the dynamic perceptual history preceding a neutral target face gives rise to an involuntary anticipation of how the emotional state of the agent would change next, called ‘emotional anticipation’, which in turn biases the perception of that neutral face [32]. These processes can be conceptualized as low-level emotion reading [8, 52], rather than as resulting from low-level visual processes (bottom-up).

In experiment 1, both HFA and TD participants showed a robust overshoot response bias with the last neutral expression of the joy-to-neutral videos evaluated as slightly angry and the identical last neutral expression of the anger-to-neutral videos as slightly happy. Contrary to the initial prediction, the extent of overshoot bias in HFA individuals did not differ from that in TD individuals. This finding suggested that the individuals with HFA were not impaired in emotional anticipation or, alternatively, that they were impaired in emotional anticipation but relied on other (compensatory) mechanisms. Experiment 2 showed that in the TD group, a change in the identity of the agent during the video clips removed the overshoot bias, confirming the findings in Palumbo and Jellema [32]. These results are compatible with top-down emotional anticipation, involving the integration of emotion and identity information available in the immediate perceptual history. Remarkably, the HFA group continued to show an overshoot bias, similar to their bias in experiment 1, which could not be ascribed to a failure to detect the change in identity. It is possible that the individuals with HFA failed to implicitly integrate the information about the identity with the information about the emotional expression. Experiment 3 further tested whether RM and contrast effects might have been responsible for the generation of the response bias in the HFA group by using video sequences that started and ended with the same neutral expression (loop condition). The responses of the HFA and TD participants did not differ in this condition, though subsequent explorative analyses suggested that the undershoot bias in the HFA group may be weaker than in the TD group, which would be compatible with the notion that in the HFA group, sequential contrast effects played a role. The ‘undershoot’ bias found in the TD group might reflect that the ‘ecologically valid’ brief smiles and frowns were seen as positive and negative signals directed at them, which made them evaluate the agent in a reciprocal manner.

### Emotional anticipation and alternative mechanisms

The notion that anticipation (or expectation) forms an integral part of perception and acts to shape perception is widely supported [53]. Especially when the anticipations are implicit or tacit, they can drive the perception of the current stimulus into the shape or direction the stimulus is expected to adopt in the immediate future and thereby distort the perception of the current stimulus [54, 55]. The emotional expressivity of a face, especially when it is embedded in a dynamic sequence, may automatically lead to an anticipation of what the agent’s emotional state most likely will be in the immediate future, possibly via a low-level mind-reading process underpinned by motor simulation involving mirror neuron mechanisms (MNM) [8], which then in turn affects perception. There is accumulating evidence that the MNM in the premotor cortex becomes activated not only when observing other’s bodily actions but also when observing other’s emotional facial expressions [18–21]. It has been suggested that such a motor simulation might underlie the tacit/intuitive understanding of others’ facial expressions [23], which in turn might shape the perception of those expressions. In this view, MNM activity is part of a low-level mind-reading mechanism [8, 52], not involving any cognitive inferential processes or deliberate reasoning, which enables emotional anticipation.

What mechanism could have driven the response bias in the HFA group in the identity-change condition? It is possible that in the HFA individuals a motor simulation of the observed dynamic expressions took place, which caused their bias in perceptual report, similar to what may have happened in the TD individuals. However, this motor simulation may not have been guided properly by identity information and therefore was applied indiscriminately, giving rise to a bias in perceptual report also in the identity-change condition. There are conflicting reports as to whether motor simulation is impaired in HFA [30]. Alternatively, it is possible that atypical means were employed as compensation for a failure in automatic emotional anticipation. It is well documented that individuals with HFA may adopt systemizing mechanisms (e.g. based on statistical regularities or input-operation-output relations), which typically govern the motions of non-agentive objects, to understand and anticipate others’ behaviour [56–59]. One such atypical approach may be a reliance on the low-level visual characteristic of the sequential contrast between the first and last frames, which were both well perceived as they were presented for 300 ms. Sequential contrast effects would predict an overshoot response in the identity-change task, as these effects are immune to identity. In principle, reliance on RM could form another atypical means resulting in perceptual biases. Although the presence of an overshoot response in the identity-change task is compatible with the notion of RM, the absence of an overshoot bias in the loop task seems to exclude this possibility.

It cannot be excluded though that the evaluations in the HFA group in the loop task were influenced by an altogether different mechanism, namely sensitivity to the pattern present in the video sequence. HFA individuals might be tuned to detecting specific sequences of changes in the stimuli and expect the pattern to repeat itself, in line with the notion that individuals with HFA are preferentially tuned to detect statistical regularities and contingencies [56]. Such a ‘pattern detection and extrapolation’ hypothesis would predict the loop to repeat itself, resulting in an undershoot bias. Subsequent experiments are required to examine the merits of the ‘sequential contrast’ and ‘pattern extrapolation’ hypotheses further in the HFA group.

Could the atypical behaviour of the HFA group have been caused by one or more (visual) difficulties characteristic for this neurodevelopmental disorder? The finding that the TD and HFA groups performed very similarly in experiment 1, and in the control conditions of experiments 2 and 3, with equally sized overshoot response biases, speaks against a contribution of such factors to the judgements. Possible impairments in visual attention and visual working memory [12], in visual perception of coherent motion [60] and in global processing [61] are therefore all unlikely contributors. Recognition of emotional facial expressions may be impaired in HFA, but recognition of prototypical happy and angry expressions is intact [62]. This is supported by the finding of very similar scores in the TD and HFA groups in the calibration phase at the start of the experiments, which also excludes the possibility that the HFA individuals might have been impaired in distinguishing between faint facial expressions of joy and anger [63].

Future studies could, in addition to investigate the role played by sensitivity to the pattern present in the video sequence in bringing about the perceptual distortions in the HFA group, also further examine the role of motor simulation in bringing about the perceptual distortions. This could be done, for example, by disrupting activity in (premotor) mirror mechanisms using transcranial magnetic stimulation. Furthermore, it would be useful to examine whether individuals with ASD having more severe symptoms than those with HFA might be even more prone to apply alternative strategies to try and make sense of other people’s emotional states from social cues.

## Conclusions

TD individuals are equipped with an automatic and mandatory mechanism, which allows them to implicitly anticipate how the emotional state of others may change on the basis of the immediate perceptual history. The current findings suggested that individuals with HFA may not rely on such an emotional anticipation mechanism, but may use alternative mechanisms.

Emotional anticipation may be underpinned by motor simulation. However, whether or not motor simulation is compromised in ASD is still debated [30, 64]. A compromised implicit and automatic anticipation mechanism would undoubtedly contribute to problems in navigating the social world, which characterize HFA.

## Abbreviations

ADOS: Autism Diagnostic Observation Schedule
AQ: Autism Spectrum Quotient
ASD: autism spectrum disorder
DSM: Diagnostic and Statistical Manual of Mental Disorders
HFA: high-functioning autism
ICD-10: International Statistical Classification of Diseases and Related Health Problems
IQ: intelligence quotient
MNM: mirror neuron mechanism
RM: representational momentum
TD: typical development
ToM: theory of mind

## References

[1] Jellema T, Lorteije J, van Rijn S, van’t Wout M, de Haan E, van Engeland H (2009). Involuntary interpretation of social cues is compromised in autism spectrum disorders. Autism Res..

[2] Senju A, Southgate V, White S, Frith U (2009). Mindblind eyes: an absence of spontaneous theory of mind in Asperger syndrome. Science..

[3] Rutter M (1978). Diagnosis and definition of childhood autism. J Autism Child Schiz..

[4] World Health Organization. ICD-10: International Statistical Classification of Diseases and Related Health Problems (10th Rev. ed.). New York: World Health Organization; 2008.

[5] American Psychiatric Association. Diagnostic and Statistical Manual of Mental Disorders (5th ed.). Washington, DC: Author; 2013.

[6] McPartland J, Klin A (2006). Asperger syndrome. Adolesc Med Clin N Am..

[7] Gallese V (2006). Intentional attunement: a neurophysiological perspective on social cognition and its disruption in autism. Brain Res..

[8] Goldman AI (2006). Simulating minds: the philosophy, psychology and neuroscience of mindreading.

[9] Frith U, Frith U (1991). Asperger and his syndrome. Autism and Asperger syndrome.

[10] Baron-Cohen S (1995). Mindblindness: an essay on autism and theory of mind.

[11] Baron-Cohen S, Campbell R, Karmiloff-Smith A, Grant J, Walker J (1995). Are children with autism blind to the mentalistic significance of the eyes. Brit J Dev Psychol..

[12] Russell J (1998). Autism as an executive disorder.

[13] Kuzmanovic B, Schilbach L, Lehnhardt FG, Bente G, Vogeley K (2011). A matter of words: impact of verbal and nonverbal information on impression formation in high-functioning autism. Res Autism Spect Dis..

[14] Blakemore SJ, Decety J (2001). From the perception of action to the understanding of intention. Nat Rev Neurosci..

[15] Jellema T, Perrett DI (2003). Perceptual history influences neural responses to face and body postures. J Cognitive Neurosci..

[16] Jellema T, Perrett DI (2003). Cells in monkey STS responsive to articulated body motions and consequent static posture: a case of implied motion?. Neuropsychologia..

[17] Kilner JM, Neal A, Weiskopf N, Friston KJ, Frith CD (2009). Evidence of mirror neurons in human inferior frontal gyrus. J Neurosci..

[18] Bastiaansen JA, Thioux M, Keysers C (2009). Evidence for mirror systems in emotions. Philos T Roy Soc Lon B Biol Sci.

[19] Carr L, Iacoboni M, Dubeau MC, Mazziotta JC, Lenzi GL (2003). Neural mechanisms of empathy in humans: a relay from neural systems for imitation to limbic areas. Proc Natl Acad Sci U S A..

[20] Leslie KR, Johnson-Frey SH, Grafton ST (2004). Functional imaging of face and hand imitation: towards a motor theory of empathy. Neuroimage..

[21] Moore A, Gorodnitskya I, Pineda J (2012). EEG mu component responses to viewing emotional faces. Behav Brain Rese..

[22] Gallese V, Fadiga L, Fogassi L, Rizzolatti G (1996). Action recognition in the premotor cortex. Brain..

[23] Rizzolatti G, Craighero L (2004). The mirror-neuron system. Annu Rev Neurosci..

[24] Rizzolatti G, Fabbri-Destro M, Cattaneo L (2009). Mirror neurons: from discovery to autism. Exp Brain Res..

[25] Fogassi L, Ferrari PF, Gesierich B, Rozzi S, Chersi F, Rizzolatti G (2005). Parietal lobe: from action organization to intention understanding. Science..

[26] Cattaneo L, Fabbri-Destro M, Boria S, Pieraccini C, Monti A, Cossu G (2007). Impairment of actions chains in autism and its possible role in intention understanding. P Natl Acad Sci-Biol..

[27] Hudson M, Burnett HG, Jellema T (2012). Anticipating action intentions in autism spectrum disorder. J Autism Dev Disord..

[28] Zalla T, Labruyere N, Clement A, Georgieff N (2010). Predicting ensuing actions in children and adolescents with autism spectrum disorders. Exp Brain Res..

[29] Dapretto M, Davies MS, Pfeifer JH, Scott AA, Sigman M, Bookheimer SY (2006). Understanding emotions in others: mirror neuron dysfunction in children with autism spectrum disorders. Nat Neurosci..

[30] Hamilton AFD (2009). Goals, intentions and mental states: challenges for theories of autism. J Child Psychol Psyc..

[31] Jellema T, Pecchinenda A, Palumbo L, Tan EG (2011). Biases in the perception and affective valence of neutral facial expressions induced by the immediate perceptual history. Vis Cogn..

[32] Palumbo L, Jellema T (2013). Beyond face value: does involuntary emotional anticipation shape the perception of dynamic facial expressions?. Plos One..

[33] Yoshikawa S, Sato W (2008). Dynamic expressions of emotion induce representational momentum. Cogn Affect Behav Ne..

[34] Suzuki S, Cavanagh P (1998). A shape-contrast effect for briefly presented stimuli. J Exp Psychol Human..

[35] Tanaka-Matsumi J, Attivissimo D, Nelson S, D’urso T (1995). Context effects on the judgment of basic emotions in the face. Motiv Emotion..

[36] Thayer S (1980). The effect of expression sequence and expressor identity on judgments of intensity of facial expression. J Nonverbal Behav..

[37] Fox CJ, Barton JJ (2007). What is adapted in face adaptation? The neural representations of expression in the human visual system. Brain Res..

[38] Hsu SM, Young AW (2004). Adaptation effects in facial expression recognition. Vis Cogn..

[39] Freyd JJ, Finke RA (1984). Representational momentum. J Exp Psychol Learn..

[40] Teufel C, Fletcher PC, Davis G (2010). Seeing other minds: attributed mental states influence perception. Trends Cogn Sci..

[41] Ashwin C, Hietanen JK, Baron-Cohen S (2015). Atypical integration of social cues for orienting to gaze direction in adults with autism. Mol Autism..

[42] American Psychiatric Association. Diagnostic and Statistical Manual of Mental Disorders (4th edition-TR ed.). Washington, DC: Author; 2000.

[43] Lord C, Rutter M, DiLavore PC, Risi S (1999). Autism Diagnostic Observation Schedule.

[44] Baron-Cohen S, Wheelwright S, Hill J, Raste Y, Plumb I (2001). The ‘Reading the Mind in the Eyes’ Test revised version: a study with normal adults, and adults with Asperger syndrome or high-functioning autism. J Child Psychol Psyc..

[45] Wechsler D (1997). WAIS-III administration and scoring manual.

[46] Ekman P, Friesen WV (1976). Pictures of facial affect.

[47] Perrett DI, May KA, Yoshikawa S (1994). Facial shape and judgements of female attractiveness. Nature..

[48] Uono S, Sato W, Toichi M (2010). Brief report: representational momentum for dynamic facial expressions in pervasive developmental disorder. J Autism Dev Disord..

[49] Behrmann M, Thomas C, Humphreys K (2006). Seeing it differently: visual processing in autism. Trends Cogn Sci..

[50] Freyd JJ (1987). Dynamic mental representations. Psychol Rev..

[51] Jones BC, DeBruine LM, Little AC, Conway CA, Feinburg DR (2006). Integrating gaze direction and expression in preferences for attractive faces. Psychol Sci..

[52] Goldman AI, Sripada CS (2005). Simulationist models of face-based emotion recognition. Cognition..

[53] Clark A (2013). Whatever next? Predictive brains, situated agents, and the future of cognitive science. Behav Brain Sci..

[54] Hudson M, Liu CH, Jellema T (2009). Anticipating intentional actions: the effect of eye gaze direction on the judgement of head rotation. Cognition..

[55] Hudson M, Jellema T (2011). Resolving ambiguous behavioral intentions by means of involuntary prioritization of gaze processing. Emotion..

[56] Baron-Cohen S (2002). The extreme male brain theory of autism. Trends Cogn Sci..

[57] Jellema T, Perrett DI, Prinz W, Hommel B (2002). Coding of visible and hidden actions. Common mechanisms in perception and action, attention and performance.

[58] Ristic J, Mottron L, Friesen CK, Iarocci G, Burack JA, Kingstone A (2005). Eyes are special but not for everyone: the case of autism. Cogn Brain Res..

[59] Jellema T, Perrett DI, Dunbar RIM, Barrett L (2012). Neural pathways of social cognition. The Oxford handbook of evolutionary psychology.

[60] Kaiser M, Shiffrar M (2009). The visual perception of motion by observers with autism spectrum disorder: a review and synthesis. Psychon B Rev..

[61] Happe´ F, Frith U. The weak coherence account: detail-focused cognitive style in autism spectrum disorders. J Autism Dev Disord. 2006;36:5–25. 10.1007/s10803-005-0039-0 16450045

[62] Uljarevic M, Hamilton A (2012). Recognition of emotions in autism: a formal meta-analysis. J Autism Dev Disord.

[63] Law Smith MJ, Montagne B, Perrett MG, Gallagher L (2010). Detecting subtle facial emotion recognition deficits in high-functioning autism using dynamic stimuli of varying intensities. Neuropsychologia..

[64] Southgate V, Gergely G, Csibra G, Pineda JA (2010). Does the mirror neuron system and its impairment explain human imitation and autism?. The role of mirroring processes in social cognition.

